# 

**Published:** 1892-08-27

**Authors:** 


**Notices of Births, Deaths, and Marriages,** are inserted in this column at a charge of 2s. 6d. for an announcement not exceeding four lines.

### NOTICE TO CORRESPONDENTS.

All MS., Letters, Books for Review, and other matters intended for the Editor should be addressed The Editor, The Lodge, Porchester Square, London, W.

The Editor cannot undertake to return rejected M.S., even when accompanied by stamped directed envelope.

**Notice to Advertisers and Subscribers.**

All Advertisements, Orders for Copies of The Hospital, and Business Communications should be addressed to The Publisher, *not to the Editor*, The Hospital, 140, Strand, W.C.

The Cost of Subscription, post free, is as follows:—Per week, 2½d.; per quarter, 2s. 6d.; per half-year, 5s.; per annum, 8s. 6d. Foreign:—Per annum, 10s. 8d.; payable, in all cases, in advance.

**"Burdett's Hospital Annual," 1891—1892.**

Third year of publication. 600 pp. Boards, gilt lettering. A most complete guide to British and Colonial Hospitals, Dispensaries, Nursing and Convalescent Institutions, and Asylums. Price 3s. 6d. Published by The Scientific Press (Limited), 140, Strand, W.C.

**NOTICE.—The Index for Vol. XI. (ending March, 1892) is on sale at the Office of this Journal, price 2½d., post free.**

### Scraps and Gleanings.

The sum of £108 8s. 2½d. collected on Hospital Saturday at Dover did not include the foreign money contributed.

Surgeon Colonel F. Welch has been appointed Principal Medical Officer on the staff of the Western Military District.

Lord Rosebery has intimated his attention of building a new Town Hall for Queensferry, in memory of his deceased wife.

M. Jovo Krsmanovics, a millionaire, and one of the most prominent merchants in Servia, has died at Belgrade, leaving a considerable sum in charities.

The death is announced in Dublin of ex-Chief Justice May, who was both Solicitor-General and Attorney-General under Mr. Disraeli. He resigned his position as Lord Chief Justice in 1887.

It was reported at a meeting of the Mansion House Committee of the fund for the relief of the sufferers by the recent hurricane in Mauritius that of £12,000 subscribed £11,522 had already been sent to Mauritius.

Princess Beatrice, who recently obtained the first-aid certificate of the St. John Ambulance Association, has become the President of a new "Deeside" centre, to include Balmoral, Craithie, and neighbouring places.

At a special meeting of the directors of the Brechin Infirmary, it was announced that the following legacies, free of duty, had been received, one from the late Ex Provost W. Duncan, Esq., for £2,000; and another of £200 from the late Mrs. Douglas.

The Court Granite City, Ancient Order of Foresters, Aberdeen, have agreed that members of the Juvenile Court should on "Hospital Day" be sent to different parts of the town, armed with money boxes in which to collect contributions for the Sick Children's Hospital.

Mr. Alfred Rimmer, of Chester, who collaborated with Dean Howson on "Ancient English Streets and Homesteads," and who wrote a description of the early homes of Prince Albert, has received from the Treasury a cheque for £100 from the Queen's Bounty Fund.

An army pensioner, named Daniel Lyons, has recently died at the great age of 103. Deceased, who lived near Castleisland, took part in several engagements in the Peninsular campaign, in which he was wounded in the left hand. He had enjoyed his pension for 78 years.

The Queen has recently added to her valuable collection of literary treasures at Windsor Castle a very old manuscript relating to Mary Queen of Scots and a hymn in the handwriting of Queen Adelaide. Her Majesty purchased both manuscripts from Miss Millard, of Teddington.

At Arnold, near Nottingham, a lad named Hunt, with two other boys, was driving a horse down a lane, when one of them struck the animal with a whip. The horse, which was heavily shod, kicked, striking Hunt on the head, inflicting such terrible injuries that he died within a few minutes.

The Seamen's Hospital Society (late Dreadnought) has recently benefited to the extent of £120 13s. through the kindness of the directors of the P. and O. Company, in throwing open to the public (at a small entrance fee) the latest addition to the company's fleet, the magnificent steamer *Himalaya*.

The death of Dr. John James Drysdale, the Homœopathic Physician is reported at the age of 75. Dr. Drysdale was the son of Sir William Drysdale, formerly Lord Provost of Edinburgh, and practised in Liverpool for 45 years. He was the author of several scientific works, and president of numerous scientific societies.

The sum of £50, on the recommendation of Mr. Balfour, has been paid out of the Royal Bounty to Mr. W. H. Escritt, the working-man poet of Guildford, the author of a tuneful volume entitled "Lyrics for the Crowd." The gift came as a welcome surprise, as Mr. Escritt has been prevented by failing health from following his ordinary avocation of a gunsmith.

A serious outbreak of scarlet fever has occurred among the Irish farm labourers employed at Kirkby and Melting, near Liverpool. The relieving officers of the West Derby and Ormskirk Unions decline to admit these patients into the workhouse hospitals. Unless something is speedily done, it is feared there will be an epidemic, as some hundreds of labourers are huddled together.

The Bank Holiday Collection at Marlow Lock, in aid of Charing Cross Hospital, amounted to £9 10s. 3½d. This sum was handed over to the Funds of Charing Cross Hospital, in recognition of Mr. J. Astley Bloxam's services to the parishioners of Little Marlow. Mr. J. A. Bloxam, F.R.C.S., who resides at Little Marlow, and is frequently to be seen on the river in the district, is Senior Surgeon at this hospital.

An epidemic of small-pox has broken out at Warrington, and is of such an alarming character that the local authorities met on Sunday night to consider what steps should be taken to prevent its spread. It was decided to erect a tent hospital outside the town, in order that the patients, of whom there are several already, may be properly isolated. One death has occurred. The disease is also spreading at Chesterfield.

At a meeting of the Waifs and Strays Society, the Committee acknowledged a gift of £1,000 for the new St. Nicholas Home for Crippled Children from Miss Anderdon. It was reported that a lady had offered £100 if nine others would give a like sum, and another had promised £25 if 19 others would give a similar donation towards the alterations, furnishing stock, and implements necessary for establishing the Walsham Home.

The Farringdon Cottage Hospital, which has been erected through the munificent liberality of Mr. William Dundas, of the Elms, Farringdon, was opened at the end of last month. The hospital consists of two wards, containing three beds each, two accident wards, matron's and servants' rooms, and all other necessary accommodation. Provision has been made for the addition, at some future time, of an operating-room.

At the annual meeting of the Huddersfield Infirmary the Chairman remarked that the workpeople's subscriptions would, he believed, ultimately amount to £1,000. The policemen have been embraced in their scheme, and the infirmary will probably receive from £15 to £20 from that source. The £1,000 given by Mr. W. Brooke for the purpose of sending convalescents to Buxton and Southport had proved a very great boon, and many had benefited by it.

### THE STUDENT OF MEDICINE.

[All communications for this Supplement should be addressed to The Editor of The Hospital, at 140, Strand. with the words. "Students' Column," written in the left hand top corner of the envelope.]

### APPOINTMENTS.

Austen, Harold, M.B., B.S.Lond., M.R.C.S., appointed Assistant Medical Officer to the Western Hospital, Fulham, S.W. Dove, Miss Emily L., M.B.Lond., appointed Resident Clinical Assistant to the Holloway Sanatorium Hospital for the Insane, Virginia Water. Ewart, C. Theodore, M.D., M.Ch., appointed Senior Assistant Medical Officer to the Female Department of the London County Asylum, Colney Hatch. Gibson, E. Valentine, M.D.Edin., appointed Superintendent and Resident Medical Officer, Victoria Infirmary, Glasgow. Gow, William J., M.D.Lond., M.R.C.P., appointed Obstetric Physician to the Royal Hospital for Children and Women, Waterloo Bridge Road. Hopkins, G. Herbert, M.R.C.S.Eng., L.R.C.P.Lond., appointed Honorary Medical Officer and Ophthalmic Surgeon to the Swansea and South Wales Institution for the Blind. Kauffmann, O. J., M.D.Univ.Lond., M.R.C.P.Lond., M.R.C.S., appointed Physician to the Out-patient Department of the Queen's Hospital, Birmingham. Macalister, Charles J., M.B.Edin., M.R.C.P.Lond., appointed Honorary Physician to the Liverpool Stanley Hospital. Newbolt, G. Palmerston, M.B.Durh., F.R.C.S.Eng., appointed Honorary Surgeon to the Stanley Hospital, Liverpool. Rees, J. L., L.R.C.P.Lond., M.R.C.S., appointed Senior Resident Surgeon to the Royal Sea Bathing Infirmary for Scrofula, Margate. Sandford, Arthur William, M.D., R.U.I., appointed Ophthalmic Surgeon to the South Infirmary, Cork. Simmens, Harold, L.R.C.P.Lond., M.R.C.S.Eng., appointed Assistant Medical Officer to the Workhouse and Infirmary of the Fulham Union. Thomson, Ritchie G., M.B., C.M.Edin., appointed Assistant Surgeon to the Royal Infirmary, Glasgow. Edie, Robert, M.B.C.M. of Oxford, has been appointed House Surgeon to the Manchester Royal Eye Hospital.

### VACANCIES.

The following vacancies are announced this week, the amount of salary in each case being given after the name of the institution:

### Resident Medical Officers.

Bedford General Infirmary, Matron—Salary £70 per annum, with board, &c.

Central London Opthalmic Hospital, House Surgeon.

City of London Hospital for Diseases of the Chest, Victoria Park, E., House-Physician for six months, commencing October 1st.

Eday, Orkney—Resident Medical Officer—Salary £70 per annum, with practice.

General Hospital, Birmingham, Assistant House Surgeon. Appointment for six months, with board, &c.

Jaffray Suburban Branch of the Birmingham General Hospital, Gravelly Hill, Medical and Surgical Officer.

Kent and Canterbury Hospital, House Surgeon; must be registered medical practitioner, and unmarried—Salary, £90 first year, with board, rising to £100 second year.

London Fever Hospital, Assistant Medical Officer.

Nottingham Borough Asylum, Second Assistant Medical Officer, unmarried—Salary, £100 per annum, with board, &c.

Royal Surrey County Hospital, House Surgeon—Salary £80 per annum.

### Non-Resident Medical Officers.

Abercarn Local Board, Medical Officer for the District—Salary £40 per annum.

London County Council, Assistant Medical Officer of Health, not less than 26 or more than 40 years of age—Salary £300 per annum, rising by annual increments of £50 until it reaches £600 a year.

Metropolitan Hospital, Dispenser.

Moville Dispensary, District Inishowen, Ireland, Medical Officer—Salary £120 per annum, exclusive of registration and vaccination fees.

St. Mary's Hospital, Paddington, W., Physician, Accoucheur.

University of Glasgow, Assistant Examiner in Medicine—Annual fee for Examinership is £30.

### PASS LISTS.

### Society of Apothecaries of London.

At the recent examinations for the prizes given annually by the Society of Apothecaries, the successful candidates were:

In Botany.—1. E. G. D. Drury, student of St. Bartholomew's Hospital, a gold medal. 2. S. B. Atkinson, student of St. Bartholomew's Hospital, a silver medal and a book.

In Materia Medica and Pharmacy.—1. R. H. Norman, student of the Westminster Hospital, a gold medal. 2. W. D. Knocker, student of St. Thomas's Hospital, a silver medal.

